# Supplementary material for: Development and external validation of machine learning algorithms for postnatal gestational age estimation using clinical data and metabolomic markers
Source: PLoS One. 2023 Mar 6;18(3):e0281074. doi: 10.1371/journal.pone.0281074 (PMC9987787; doi:10.1371/journal.pone.0281074)
Supplement: S1 File — (DOCX) [file pone.0281074.s001.docx]

**Title: Development and external validation of machine learning algorithms for postnatal gestational age estimation using clinical data and metabolomic markers**

**Authors and affiliations**

Steven Hawken*, PhD Clinical Epidemiology Program, Ottawa Hospital Research Institute, 501 Smyth Rd, Ottawa, Canada, K1H 8L6; shawken@ohri.ca

Robin Ducharme, MSc Clinical Epidemiology Program, Ottawa Hospital Research Institute, 501 Smyth Rd, Ottawa, Canada, K1H 8L6; roducharme@ohri.ca

Malia S.Q. Murphy, PhD Clinical Epidemiology Program, Ottawa Hospital Research Institute, 501 Smyth Rd, Ottawa, Canada, K1H 8L6; malmurphy@ohri.ca

Brieanne Olibris, MPH Clinical Epidemiology Program, Ottawa Hospital Research Institute, 501 Smyth Rd, Ottawa, Canada, K1H 8L6; bolibris@ohri.ca

A. Brianne Bota, PhD Clinical Epidemiology Program, Ottawa Hospital Research Institute, 1053 Carling Ave, Ottawa, Canada, K1Y 4E9; bbota@ohri.ca

Lindsay A. Wilson, MSc Clinical Epidemiology Program, Ottawa Hospital Research Institute, 1053 Carling Ave, Ottawa, Canada, K1Y 4E9; liwilson@ohri.ca

Wei Cheng, PhD Clinical Epidemiology Program, Ottawa Hospital Research Institute, Ottawa, Canada; wei.cheng.stat@gmail.com

Julian Little, PhD School of Epidemiology and Public Health, University of Ottawa, 600 Peter Morand Crescent, Ottawa, Canada, K1G 5Z3; jlittle@uottawa.ca

Beth K. Potter, PhD School of Epidemiology and Public Health, University of Ottawa, 600 Peter Morand Crescent, Ottawa, Canada, K1G 5Z3; Beth.Potter@uottawa.ca

Kathryn M. Denize, MSc Newborn Screening Ontario, Children’s Hospital of Eastern Ontario, 415 Smyth Rd, Ottawa, Canada, K1H 5A4; kdenize@cheo.on.ca

Monica Lamoureux, MSc Newborn Screening Ontario, Children’s Hospital of Eastern Ontario, 415 Smyth Rd, Ottawa, Canada, K1H 5A4; MoLamoureux@cheo.on.ca

Matthew Henderson, PhD Newborn Screening Ontario, Children’s Hospital of Eastern Ontario, 415 Smyth Rd, Ottawa, Canada, K1H 5A4; MHenderson@cheo.on.ca

Katelyn J. Rittenhouse, MD University of North Carolina at Chapel Hill, Chapel Hill, USA; [Katelyn.Rittenhouse@unchealth.unc.edu](mailto:Katelyn.Rittenhouse@unchealth.unc.edu)

Joan T. Price, MD University of North Carolina at Chapel Hill, Chapel Hill, USA, 27599; [joni@email.unc.edu](mailto:joni@email.unc.edu)

Humphrey Mwape UNC Global Projects Zambia, Lusaka, Zambia; Humphrey.mwape@unclusaka.org

Bellington Vwalika, MBChB, MPH Department of Obstetrics and Gynaecology, University of Zambia School of Medicine, Lusaka, Zambia; vwalikab@gmail.com

Patrick Musonda, PhD Department of Medical Statistics, University of Zambia College of Public Health; Lusaka, Zambia; pmuzho@hotmail.com

Jesmin Pervin, MPH International Centre for Diarrhoeal Disease Research, 68 Shaheed Tajuddin Ahmed Sarani, Mohakhali, Dhaka, 1212, Bangladesh; jpervin@icddrb.org

AK Azad Chowdhury Dhaka Shishu (Children) Hospital, Sher-e-Bangla Nagar, Dhaka, 1207, Bangladesh; childcare1952@gmail.com

Anisur Rahman, PhD International Centre for Diarrhoeal Disease Research, 68 Shaheed Tajuddin Ahmed Sarani, Mohakhali, Dhaka, 1212, Bangladesh; arahman@icddrb.org

Pranesh Chakraborty, MD, FRCPC, FCCMG

Newborn Screening Ontario, Children’s Hospital of Eastern Ontario, 415 Smyth Rd, Ottawa, Canada, K1H 5A4; PChakraborty@cheo.on.ca

Jeffrey S.A. Stringer, MD Department of Obstetrics and Gynecology, University of North Carolina at Chapel Hill, Chapel Hill, USA, 27599; jeffrey_stringer@med.unc.edu

Kumanan Wilson, MD FRCPC Clinical Epidemiology Program, Ottawa Hospital Research Institute, 1053 Carling Ave, Ottawa, Canada, K1Y 4E9; kwilson@ohri.ca

***Corresponding Author:** Dr. Steven Hawken

Ottawa Hospital Research Institute

Centre for Practice Changing Research

501 Smyth Rd, Box 201B

Ottawa, ON Canada

[shawken@ohri.ca](mailto:shawken@ohri.ca)

613-737-8899 x10672

**S1 Supporting Information: Supplemental study methods and results**

**Supplemental Methods**

**Sample collection and analysis**

Four blood spots of approximately 75 µL each were collected from the umbilical cord (Zambia and Bangladesh cohorts only) within 30 minutes of delivery and by newborn heel prick (preferably 24 to 72 hours following delivery). The vast majority of samples from Ontario were collected in the 24-72 hour window. For the Zambia and Bangladesh cohorts, the protocol was for samples to be collected between 24 to 72 hours post-delivery, but mothers often returned home with their newborn within a few hours of birth, so staff were instructed to collect heel prick samples as late as possible before mothers and infants returned home which was often before 24 hours had elapsed. All samples were collected onto Whatman 903 filter paper, air-dried, and shipped to Newborn Screening Ontario (NSO), a provincial newborn screening laboratory in Ottawa, Canada. Nine 32 mm diameter samples were punched from each dried blood spot sample and analyzed for the following analytes (detailed in Table 1): Hemoglobin profiles determined by high performance liquid chromatography on a Bio Rad Variant^TM^ nbs system; neonatal 17-Hydroxyprogesterone (17-OHP), thyroid stimulating hormone (TSH) and immunoreactive trypsinogen (IRT) measured using a PerkinElmer AutoDELFIA® Immunoassays; a panel of 12 amino acids and 31 acylcarnitines measured by electrospray ionization tandem mass spectrometry (Waters TQD); and biotinidase and galactose-1-phosphate uridyltransferase levels measured using the Astoria-Pacific SPOTCHECK® Pro system.

**Data preparation**

In preparation for use in modeling, newborn screening analytes were winsorized using an adapted “Tukey Fence” approach.^1^ For each analyte, this involves assigning values more than three interquartile ranges above the third quartile (the upper Tukey fence) or below the first quartile (the lower Tukey fence), to the Tukey fence value, or the smallest/largest observed value in the dataset, whichever was the least extreme. This approach preserves much of the “extremeness” of outliers but prevents extreme values from disproportionately impacting model building and parameter estimation. The majority of measured analytes exhibit strongly right-skewed distributions, which was addressed though natural log transformation, which also stabilizes the variance, reducing the impact of heteroskedasticity. Finally, both analyte levels and birth weight values were normalized by subtracting the mean and dividing by the square root of the standard deviation for each variable (pareto scaling) ^2,3^, which centers all predictors to have a mean of zero, and scales them to reduce the impact of variations in dispersion of individual analytes across cohorts.

**Model Development in the Ontario, Canada model derivation cohort**

Briefly, a cohort of 159,131 infants born between January 2012 and December 2014 was derived from the BORN Ontario birth registry which included all required predictors as well as reference standard GA measured by confirmed 1^st^ trimester gestational dating ultrasound.

For Models 1 and 3, birth weight was modeled using a restricted cubic spline with five knots to allow for non-linearity of the association of birth weight with gestational age. For Models 2 and 3, we included all pre-specified covariate main effects in the models, however we additionally identified the most predictive analytes using the metric of generalized partial Spearman correlation that detects non-linear and non-monotonic associations with GA, mutually adjusted for all other analytes and clinical covariates. Based on this partial Spearman correlation analysis there were seven analyte covariates that had distinctly stronger partial correlations with GA compared to all others. These seven analytes were modeled using restricted cubic splines with 5 knots. These were (in order of strength of partial spearman correlation): fetal-to-adult hemoglobin ratio, 17-OHP, C4DC, TYR, ALA, C5, and C5DC. For birthweight, and the seven strongest analyte predictors, knot placement was at the 5th, 27.5th, 50th, 72.5th, and 95th percentiles based on the Ontario population distribution of these analytes ^4^.

For Model 1, all covariates and pairwise interactions were included in the model without variable selection or regularization. For Models 2 and 3 we employed Elastic Net regularization, which employs two forms of penalization (called L1 and L2 regularization) to simultaneously estimate regression coefficients while also shrinking them towards zero to penalize the increase in model complexity from each additional term included in the model.^5^. The Elastic Net regression methodology allows models to be fit with a large number of predictors, even models where the number of predictors exceeds the number of observations (p>>n), and provides strong protection against overfitting, and against the instability inherent in fitting models with a large number of predictors relative to the number of observations available for model fitting ^5,6^.

Given the strong imbalance in preterm gestation versus term and post-term gestation in our population cohort of infants, we used a weighting scheme to reduce the dominance of term infants in model development. We used an ad hoc approach to developing the weighting scheme in which preterm and post-term infants were upweighted in model training. We did not strictly derive the weights to give equal weight to all gestational ages, as when we did this, overall model performance was negatively impacted. We iteratively increased the weights in preterm and post-term infants until we identified a threshold where the internal validation MSE in preterm/post-term infants was maximized but MSE in term infants hadn’t declined substantially. These weights were developed in earlier model building studies, and the same weighting scheme was used in the current study. Hence development of weights was not undertaken in any of the model training, validation or testing data from the current study. The weights used were as follows:

22-33 weeks weight=10

34-35 weeks weight=5

36-37 weeks weight=2

38-40 weeks weight=1

41+ weeks weight=2

**Imputation and Bootstrap Confidence Intervals**

A small proportion of analyte values were missing for subjects in the Zambia and Bangladesh cohorts, due to issues such as sample quality, insufficient sample quantity to complete all assays, degradation during shipping. Due to the limited sample size available from the external validation cohorts, and the fact that only a very small subset of analyte values were missing in any one individual, missing analyte values in external validation cohorts were imputed. Given the planned use of multiple imputation, and the fact that parametric standard error estimates were not readily calculable for our validation performance metrics, valid parametric confidence intervals could not be calculated. We therefore elected to calculate bootstrap percentile confidence intervals based on the 2.5th and 97.5th percentiles of performance metrics calculated from bootstrap samples. Simultaneous imputation of missing covariate data (which is usually accomplished through multiply imputing the dataset and then adjusting standard estimates based on the inter-imputation variance across the multiple imputations), and generation of bootstrap replicates for the same dataset, creates some novel methodological challenges in appropriately incorporating the multiple sources of variation. This is an active area of current research. Several candidate approaches were evaluated in a recently published simulation study ^7^. Based on that study, we chose the recommended confidence interval method that demonstrated the most accurate coverage probability. This method involves single imputation nested within the bootstrap percentile method, with additional random variation added to each imputation to reflect the uncertainty of the imputation step. Single imputations were conducted using a predictive mean matching algorithm ^4,8^ using non-missing analyte values for all subjects in the external cohorts. Birthweight and GA were not used in the imputation process to avoid biasing the validation results. The combination of bootstrap resampling and imputation steps was programmed in R and used the aregImpute function which implements the required predictive mean matching algorithm developed and refined by Harrell and van Buuren ^4,8^. Based on this method, we generated 2000 singly imputed bootstrap replicates for both the Zambia and Bangladesh cohorts.

For the Ontario, Canada cohort, a small proportion of observations also had missing analyte values. However the proportion of missingness was extremely low and the available sample size large enough, that we elected to utilize complete cases in model development and internal validation. Therefore we were able to calculate standard bootstrap percentile confidence intervals for performance metrics for the Ontario internal validation in the independent test cohort.

**Validation of GA estimation models**

Our external validation protocol has been reported elsewhere ^9^. We applied the identical approach in validating models internally (in Ontario test cohort) and externally (Zambia and Bangladesh cohorts).

Final Ontario regression model equations were used to calculate an estimated gestational age in the Ontario test cohort and in the Zambia and Bangladesh cohorts. For each infant, model performance was assessed by comparing the estimated gestational age from the model to the ultrasound-derived gestational age and calculating the difference between the model estimate and ultrasound-based estimate. Agreement was measured by calculating the mean absolute error (MAE) measured in weeks (the average of the absolute values of the model vs. ultrasound difference across all observations). Lower MAE reflects higher accuracy. We also calculated the percentage of infants with gestational ages correctly estimated within 7 days of ultrasound-based gestational age. We assessed model performance overall and in important subgroups: preterm birth (<37 weeks gestation), and small-for-gestational age (SGA10 and SGA3). SGA10 and SGA3 were designated in cases where birth weight was below the 10^th^ and 3^rd^ percentile within categories of gestational week at delivery and infant sex, respectively. The percentiles were calculated based on INTERGROWTH-21 sex and gestational age categories ^10^.

All reported performance metrics were reported as the mean, and 2.5^th^ and 97.5^th^ bootstrap confidence limits.

**Sensitivity Analysis**

**Restricted models including only analytes that are highly correlated between heel prick and cord blood samples**

We conducted an analysis of pairwise Spearman correlation between heel prick and cord blood analyte levels in a pooled cohort of infants from the Zambia and Bangladesh cohorts with both sample types. We then fit new reference models in Ontario data including only the analytes in the model with a minimum Spearman correlation coefficient of 0.5 (Model 4) and 0.3 (Model 5) between paired heel prick and cord blood samples. We then conducted a sensitivity analysis where we validated model performance in both the Zambia and Bangladesh cord blood cohorts using these restricted models. The aim of this sensitivity analysis was to investigate whether a subset of analytes, demonstrating higher agreement between heel prick and cord blood samples from the same infant, would improve the performance of models developed using heel prick data, when applied to cord blood samples.

**Supplemental Results**

**Sensitivity Analysis**

**Restricted models including only analytes that are highly correlated between heel prick and cord blood samples**

A total of 17 analytes met the threshold of minimum Spearman correlation coefficient of 0.5 between heel prick and cord blood samples from the pooled Bangladesh and Zambia cohorts (fetal-to-adult hemoglobin ratio, C4DC, C0, IRT, C3, C2, C18, C16, C4, C18_1, BIO, C8_1, GALT, Cit, C18_2, Arg and C5) and were included in Model 4. A total of 30 analytes met the threshold of minimum Spearman correlation coefficient of 0□3 between heel prick and cord blood samples in the pooled cohort (fetal-to-adult hemoglobin ratio, C4DC, C0, IRT, C3, C2, C18, C16, C4, C18_1, BIO, C8_1, GALT, Cit, C18_2, Arg, C5, Gly, C5OH, C14, Orn, C6DC, C3DC, C16_1OH, C5DC, _17OHP, C4OH, MET, C6 and Ala) and were included in Model 5. As in the primary external validation analysis, BIO, IRT and GALT were excluded from the model for the Zambia cohort due to a high number of missing values, so slightly reduced versions of Model 4 and Model 5 were applied in the Zambia cohort, with 14 and 27 analytes included in Model 4 and Model 5 respectively. When the models fit in Ontario data including sex, multiple gestation, birthweight and the restricted set of analytes were applied to the Zambia cord blood cohort, overall performance was very similar to results for the full model (Model 3) from the main results. Model 4, when applied to cord blood samples from Zambia yielded an MAE of 1.01 (0.90, 1.12) and for Model 5 the MAE was 1.04 (0.91, 1.17), compared to an MAE of 1□02 (0.90, 1.15) for Model 3 from the main results. Model 4 when applied to cord blood samples from Bangladesh, yielded an MAE of 0.95 (0.91, 0.99), and for Model 5 the MAE was 0.95 (0.90, 0.99) compared to an MAE of 0.95 (0.90, 0.99) for Model 3 from the main results. However, the restricted models did demonstrate better precision in estimating GA in cord blood from preterm infants. In Zambia, model 4 yielded an MAE of 2.49 (1.69, 3.37) and Model 5 yielded an MAE of 2.87 (1.96, 3.84), compared to Model 3 from the main analysis which had an MAE of 3.01 (2.15, 3.99). In Bangladesh, MAE was 1.67 (1.49, 1.86) for Model 4 and 1.81 (1.63, 1.99) for Model 5, compared to 1.92 (1.74, 2.11) from the main analysis.

**References**

1 Tukey JW. Exploratory data analysis, 1st edn. Reading: Addison‐Wesley Publishing Company, 1977.

2 van den Berg RA, Hoefsloot HCJ, Westerhuis JA, Smilde AK, van der Werf MJ. Centering, scaling, and transformations: Improving the biological information content of metabolomics data. *BMC Genomics* 2006; **7**. DOI:10.1186/1471-2164-7-142.

3 Eriksson L, Johansson E, Kettapeh-Wold S, Wold S. Scaling. In: Introduction to multi- and megavariate data analysis using projection methods (PCA & PLS). Umetrics, 1999: 213–25.

4 Harrell FE. Regression Modeling Strategies with Applications to Linear Models, Logistic and Ordinal Regression and Survival Analysis, 2nd edn. New York: Springer.

5 Zou H, Hastie T. Regularization and variable selection via the elastic net. *J R Stat Soc Ser B Stat Methodol* 2005. DOI:10.1111/j.1467-9868.2005.00503.x.

6 Hastie T, Tibshirani R, Friedman J. The Elements of Statistical Learning : Data Mining, Inference, and Prediction, 2nd edn. New York: Springer, 2008.

7 Brand J, van Buuren S, le Cessie S, van den Hout W. Combining multiple imputation and bootstrap in the analysis of cost-effectiveness trial data. *Stat Med* 2019. DOI:10.1002/sim.7956.

8 van Buuren S. Flexible Imputation of Missing Data, 1st edn. New York: CRC Press, 2013.

9 Murphy MSQ, Hawken S, Atkinson KM, *et al.* Postnatal gestational age estimation using newborn screening blood spots: a proposed validation protocol. *BMJ Glob Heal* 2017; **2**: e000365.

10 Villar J, Giuliani F, Fenton TR, Ohuma EO, Ismail LC, Kennedy SH. INTERGROWTH-21st very preterm size at birth reference charts. Lancet. 2016; **387**: 844–5.
