# Supplementary material for: Development and external validation of machine learning algorithms for postnatal gestational age estimation using clinical data and metabolomic markers
Source: PLoS One. 2023 Mar 6;18(3):e0281074. doi: 10.1371/journal.pone.0281074 (PMC9987787; doi:10.1371/journal.pone.0281074)
Supplement: Attachment — Submitted filename: Response to Reviewers.docx [file pone.0281074.s003.docx]

**PONE-D-22-01354**

Development and external validation of machine learning algorithms for postnatal gestational age estimation using clinical data and metabolomic markers

**Journal Requirements:**

When submitting your revision, we need you to address these additional requirements.

1. Please ensure that your manuscript meets PLOS ONE's style requirements, including those for file naming. The PLOS ONE style templates can be found at 
https://journals.plos.org/plosone/s/file?id=wjVg/PLOSOne_formatting_sample_main_body.pdf and 
<https://journals.plos.org/plosone/s/file?id=ba62/PLOSOne_formatting_sample_title_authors_affiliations.pdf>

***Author Response:* We have updated our manuscript to meet PLOS ONE’s style requirements.**

2. Please include a complete copy of PLOS’ questionnaire on inclusivity in global research in your revised manuscript. Our policy for research in this area aims to improve transparency in the reporting of research performed outside of researchers’ own country or community. The policy applies to researchers who have travelled to a different country to conduct research, research with Indigenous populations or their lands, and research on cultural artefacts. The questionnaire can also be requested at the journal’s discretion for any other submissions, even if these conditions are not met.  Please find more information on the policy and a link to download a blank copy of the questionnaire here: https://journals.plos.org/plosone/s/best-practices-in-research-reporting. Please upload a completed version of your questionnaire as Supporting Information when you resubmit your manuscript.

***Author Response:* We have uploaded a copy of the questionnaire.**

3. We note that the grant information you provided in the ‘Funding Information’ and ‘Financial Disclosure’ sections do not match.

When you resubmit, please ensure that you provide the correct grant numbers for the awards you received for your study in the ‘Funding Information’ section.

***Author Response:* We have now updated the Funding Information data to reflect what is written in our Financial Disclosure section.**

4. In your Data Availability statement, you have not specified where the minimal data set underlying the results described in your manuscript can be found. PLOS defines a study's minimal data set as the underlying data used to reach the conclusions drawn in the manuscript and any additional data required to replicate the reported study findings in their entirety. All PLOS journals require that the minimal data set be made fully available. For more information about our data policy, please see <http://journals.plos.org/plosone/s/data-availability>.

Upon re-submitting your revised manuscript, please upload your study’s minimal underlying data set as either Supporting Information files or to a stable, public repository and include the relevant URLs, DOIs, or accession numbers within your revised cover letter. For a list of acceptable repositories, please see http://journals.plos.org/plosone/s/data-availability#loc-recommended-repositories. Any potentially identifying patient information must be fully anonymized.

Important: If there are ethical or legal restrictions to sharing your data publicly, please explain these restrictions in detail. Please see our guidelines for more information on what we consider unacceptable restrictions to publicly sharing data: http://journals.plos.org/plosone/s/data-availability#loc-unacceptable-data-access-restrictions. Note that it is not acceptable for the authors to be the sole named individuals responsible for ensuring data access.

We will update your Data Availability statement to reflect the information you provide in your cover letter.

***Author Response:* We have received approval from our institutional REB and legal teams to prepare minimum de-identified datasets to be shared on a public data repository. We are currently in the process of preparing and uploading the data to Dryad and will provide the DOI upon acceptance of our manuscript.**

5. We note that you have stated that you will provide repository information for your data at acceptance. Should your manuscript be accepted for publication, we will hold it until you provide the relevant accession numbers or DOIs necessary to access your data. If you wish to make changes to your Data Availability statement, please describe these changes in your cover letter and we will update your Data Availability statement to reflect the information you provide.

***Author Response:* As mentioned above, we now have approval to share our de-identified data to a public repository and will share the DOI upon manuscript acceptance.**

6. Please note that in order to use the direct billing option the corresponding author must be affiliated with the chosen institute. Please either amend your manuscript to change the affiliation or corresponding author, or email us at plosone@plos.org with a request to remove this option.

***Author Response:* Thank you. We will email the journal to remove the option for direct billing.**

7. Please amend the manuscript submission data (via Edit Submission) to include authors Kathryn M. Denize, Monica Lamoureux, Matthew Henderson and Joan T. Price.

***Author Response:* We have added these authors to the submission data.**

8. Please upload a new copy of Figures 1-4 as the detail is not clear. Please follow the link for more information: https://blogs.plos.org/plos/2019/06/looking-good-tips-for-creating-your-plos-figures-graphics/" <https://blogs.plos.org/plos/2019/06/looking-good-tips-for-creating-your-plos-figures-graphics/>

***Author Response: We have uploaded revised versions of Figures 1 to 4.***

9. Please include captions for your Supporting Information files at the end of your manuscript, and update any in-text citations to match accordingly. Please see our Supporting Information guidelines for more information: [http://journals.plos.org/plosone/s/supporting-information.](http://journals.plos.org/plosone/s/supporting-information. )

***Author Response:* Thank you. We have added a caption and in-text citations as requested.**

5. Review Comments to the Author

Please use the space provided to explain your answers to the questions above. You may also include additional comments for the author, including concerns about dual publication, research ethics, or publication ethics. (Please upload your review as an attachment if it exceeds 20,000 characters)

Reviewer #1: Steven Hawken and colleagues presented three machine learning-based models to estimate GA shortly after birth using clinical and metabolomics data. The models were trained and internally validated with data from Ontario - Canada and externally validated with data from Zambia and Bangladesh. I found the manuscript very interesting and it is well written. However, there are some issues that the authors should address before its publication.

- In Table 3, it would be nice to add the p-value to see whether there are statistical differences between the 3 cohorts.

***Author Response:* We have focused on providing confidence intervals for the presented model performance metrics rather than explicit hypothesis testing. However, we acknowledge that p-values can provide additional information about important comparisons, beyond just comparing the overlap of confidence regions. We are in the process of developing new algorithms to generate p-values and confidence intervals for difference tests for contrasts of interest for application to future studies. However, because we are calculating uncertainty of estimates using bootstrap methods, and some comparisons are for different models used on the same cohort, and other comparisons are for the same model applied to independent cohorts, the methodology is not straightforward and we are unable to complete this for the current study since developing and testing the methodology is still a work in progress.**

- There are a couple of mistakes in Table 3: In the third column, in the birgh weight Overall row it says: 31020 grams. In addition, on the row below, there is a space missing between the mean and (SD) values.

***Author Response:* Thank you. We have made these corrections.**

- Lines 231-232: “Accuracy of estimated GA was generally lower in the external validation cohorts than in the Ontario internal validation results for the same models” à “Accuracy of estimated GA was generally lower in the external validation cohorts than in the Ontario internal validation COHORT for the same models”

***Author Response:* Thank you, we have made this correction.**

- I cannot see Tables 4 and Table 5 properly, since they are cut.

***Author Response:* We have changed the orientation of the tables to make them display properly.**

- I am unable to see the numbers on the axis of Figure 2… Can you please make the numbers bigger? Same comment applies to Figures 3 and 4.

***Author Response: We have improved the axis labelling for Figures 2,3 and 4 to make them easier to read.***

- Lines 325 – 334: Instead of giving the percentage of preterm births estimated by each model and for each cohort, it would be more interesting to give the number of infants correctly classified as preterm by the 3 models, and for the 3 cohorts, since, as stated by the authors, one of the main objectives of their work is the properly identification of premature infants at birth, in middle and low-income countries. When do so, please provide also, other quantitative measurements such as sensitivity, specificity, etc. for preterm detection,

***Author Response:* Our models were intended for estimation of continuous gestational age rather than dichotomous classification, however we agree this is useful information given that we’ve reported the preterm birth rates that would be estimated by our Models. A model optimized for classification would generally be a logistic regression, and the probability cutpoint for classification would be likely be optimized depending on the application to favor sensitivity or specificity. Therefore, in the interest of parsimony, we’ve now reported the classification accuracy and sensitivity and specificity of Model 3 in the Ontario, Bangladesh and Zambia heel prick cohorts, and acknowledged that the reported performance in Cord blood cohorts would be markedly worse than in heel prick cohorts, as would Models 1 and 2 in all cohorts as compared to Model 3.**
- Have the infants included in this study any important disease, such as, for example congenital heart disease?

***Author Response:* The Ontario cohort is population-based and includes all infants born within the specified time frame. Therefore, this cohort includes infants with and without congenital and other diseases, but we do not have information available on these diagnoses. Mothers of infants included in the external validation cohorts were enrolled into the study during pregnancy. We do not have any information on any infant diagnoses either before or after birth, so it is possible that these cohorts include infants with congenital and / or other diseases.**

- The performance of the 3 models on correctly predicting GA in preterms is quite low (Model 3: only 28.3% of the 11 preterm infants in Zambia cohort have an absolute error o equal or below to 1 week, when using heel prick samples, for example), as seen in the results provided in tables 4 and 5. Have the authors thought on ways to improve the model performance on this subset of sample? Have the authors considered the use of other sources of data together with heel trick and clinical data? What about data imputation? Can this have a negative effect on model performance when applied to preterm subset?

***Author Response:* Our investigation is part of a broader project to develop non-invasive GA estimation methods. Other groups have been looking at lens vascularity, other athropomorphic methods, DNA methylation, untargeted metabolomics etc., which have all demonstrated some promise. We have not had the opportunity to collect a larger set of these markers in the same cohort of infants, however we recognize that pooling the most promising markers identified across studies would undoubtedly provide a more accurate model, and could be the focus of future work. The main challenge would be collecting these markers in a large enough cohort of infants in the model derivation cohort. Our aim in the current study was to externally validate our best algorithms derived in a defined set of markers already collected in all newborns in Ontario, and which could be measured in our external validation cohort.**

**Imputation of analyte values would not have markedly impacted model performance, as only a very small proportion of values were actually imputed, and this imputation used an approach that harnessed the correlation among analyte levels within individuals. Similarly, imputation would be very unlikely to impact preterm model performance, because only 3 of 11 preterm infants had analyte values imputed (and 1/3 had only 1 analyte value imputed, and 2/3 had 2 analyte values imputed).**

- Please, carefully review your Supplementary material document, since there are some characters that are not displayed properly (i.e.: “Ontario data including only the analytes in the model with a minimum Spearman correlation coefficient of 0□5 (Model 4) and 0·3 (Model 5)”. Also, the decimal points used sometimes is wrong… It should be 0.91 instead of 0·91, for example.

***Author Response:* We have edited the document and replaced all incorrect characters.**

- Supplementary method; “We also calculated the percentage of infants with gestational ages correctly estimated within 7 and 14 days of ultrasound-based gestational age” I thought, according to the methods described in the main manuscript, that you calculated the percentage of infant with GA correctly estimated within 1 and 7 days, since this would correspond to an error of +/-1 week. However, GA estimated within 7 and 14 days would be +/-2 weeks…

***Author Response:* Thank you. We have corrected the text to reflect our final analysis, which calculated the percentage correctly estimated within 1 week.**
