# Supplementary material for: Development and external validation of machine learning algorithms for postnatal gestational age estimation using clinical data and metabolomic markers
Source: PLoS One. 2023 Mar 6;18(3):e0281074. doi: 10.1371/journal.pone.0281074 (PMC9987787; doi:10.1371/journal.pone.0281074)
Supplement: Attachment — Submitted filename: Response to reviewer comments.docx [file pone.0281074.s002.docx]

**Thank you for the opportunity to provide a revised version of our manuscript.**

2. In your Data Availability statement, you have not specified where the minimal data set underlying the results described in your manuscript can be found. PLOS defines a study's minimal data set as the underlying data used to reach the conclusions drawn in the manuscript and any additional data required to replicate the reported study findings in their entirety. All PLOS journals require that the minimal data set be made fully available. For more information about our data policy, please see http://journals.plos.org/plosone/s/data-

In your revised cover letter, please address the following prompts:

a) If there are ethical or legal restrictions on sharing a de-identified data set, please explain them in detail (e.g., data contain potentially sensitive information, data are owned by a third-party organization, etc.) and who has imposed them (e.g., an ethics committee). Please also provide contact information for a data access committee, ethics committee, or other institutional body to which data requests may be sent.

b) If there are no restrictions, please upload the minimal anonymized data set necessary to replicate your study findings as either Supporting Information files or to a stable, public repository and provide us with the relevant URLs, DOIs, or accession numbers. For a list of acceptable repositories, please see http://journals.plos.org/plosone/s/data-availability#loc-recommended-repositories.

We will update your Data Availability statement on your behalf to reflect the information you provide.

**We will post our minimum datasets to a public data repository; we are currently finalizing steps required by our local REB and legal teams.**

Reviewer #1: This study develops machine learning algorithms to estimate the gestational age (GA) after birth based on clinical and metabolic data. The authors validate their models internally using data from a cohort of infants born in Canada and externally using data from newborns in Zambia and Bangladesh. While it considers an interesting and important topic, addressing a number of issues can improve the manuscript.


1. This study aims to develop reliable alternatives for the estimation of GA in low and middle-income countries. However, all of the test samples were analyzed centrally in a Canadian lab. Considering the challenges of acquiring high-quality and timely lab results in lower-income countries [1], it would be helpful to discuss how uncertainty in the lab results would influence the predictions of the models.
[1] Wilson ML, et. al. Access to pathology and laboratory medicine services: a crucial gap. *Lancet*. 2018

**The authors agree that there are many barriers to implementing model GA estimations in practice, the most significant perhaps is conducting analysis in LMICs. We have included a discussion on challenges to implementing this technology in LMICs in the discussion. The discussion now reads:**

*“Implementation of our postnatal GA estimation models in LMIC settings to determine population level GA distribution was the end goal of our research, but would present many challenges. The current process involving of local sample collection and international analysis has been successful in our proof of concept, external validation and feasibility work. However, implementing the analysis pipeline locally would be ideal. Many LMIC are lacking in pathology and laboratory medicine services. Barriers include insufficient infrastructure, properly trained personnel and insufficient quality, standards and accreditation education and training for laboratory and medicine personnel. Many newborn screens use tandem mass spectrometry, which has limited availability in many LMIC settings.”*

2. The authors use Spearman’s rank correlation to evaluate the monotonic relationship between the analyte measurements from heel prick and cord blood data. Please explain why the nonlinear correlation is evaluated? Linear regression models should be linear in the parameters; are the independent variables in this study modified to fit nonlinear data? If not, how the nonlinear correlation improves the selection of parameters in the linear model?

**The approach we used was developed by Frank Harrell, a leading expert in statistical modeling and presented in his “Regression Modeling Strategies” monograph. It is not a variable screening procedure but rather an approach to allocating additional degrees of freedom to the estimation of a subset of important continuous predictors (all of which are included in the model as linear terms otherwise). No predictor has an exact linear association with the dependent variable in a regression, but in many cases a continuous covariate can be well modeled using a linear term and it would be prohibitive (and often unnecessary) to use polynomial or spline effects - which require a large number of model terms (degrees of freedom) to flexibly model a non-linear association. The reason the Spearman correlation approach was used, was to identify the strongest independent associations (linear or non-linear) among all of the predictors. Then, if a subset of continuous predictors are more strongly associated with GA, they could be modeled using splines or some other approach, while the balance of the predictors would be modeled either with linear terms for continuous variables or class/categorical terms for other predictors. Modeling all continuous covariates using spline effects would not have been feasible or necessary.**

3. Gestational age is systematically overestimated for the external cohorts, and expectedly, the models are more accurate for term infants. The authors provide several explanations, but this issue seems to most likely arise from the imbalanced training set, which primarily consists of infants born at term. The model is overfitted to the group represented in the dataset, and mostly neglects the minority group (preterm infants). While such a model might seem to be highly accurate in testing (because most of the test data are also from term infants), it often fails to identify premature infants, the most vulnerable group. Model performance for estimating the rate of preterm birth also confirms that the trained models do not adequately represent the minority group. There are several ways to deal with imbalanced datasets, e.g., under-sampling, over-sampling, and creating synthetic samples. This preprocessing step seems to be missing in this study.

**Thank you for this observation. In fact, we used an oversampling approach as you suggested, where we upweighted preterm infants in model development in order to balance out the influence of more common gestational ages (term infants) compared to increasingly preterm infants. We omitted this methodological step in the original description of statistical methods, which we have now included in the revised methods to correct this oversight:**

*“To address the large imbalance in number of term infants compared to preterm and post-term infants, we applied a weighting scheme in the regression model fitting process to balance the influence of term infants with less common gestational ages (additional details in Appendix).”*

4. It would be informative to discuss why the model performs better for heel prick samples, compared to the cord blood data. Is it because the model is trained using heel prick samples? Could it have a better performance for cord blood samples if trained using cord blood data? Is it always advantageous to use the heel prick sample instead of the cord blood sample?

**We have added additional detail to the discussion regarding the relative performance of models in cord vs. heel blood, and why we weren’t able to derive a cord blood-specific model. The discussion now reads:**

*“We validated the performance of our GA models, which were derived using heel-prick samples, in umbilical cord blood from the Zambia and Bangladesh cohorts. Estimation of GA using cord blood would provide several advantages. It would reduce the sample collection burden on staff, resulting in comparably less stress for the newborn and parents and requires less training compared to heel prick sample collection.*

*Unfortunately, when we applied our GA models to cord blood samples, we observed sharply diminished accuracy of predictions in both the Zambia and Bangladesh cohorts. There were two main reasons for this. First, our models were derived using heel prick samples. We were unable to derive cord blood-specific models as cord blood is not routinely collected in our Ontario population. Second, our investigations in paired heel and cord samples showed that only a small subset of cord blood analyte levels are closely correlated with heel prick analytes levels in the same infants, so decreased accuracy is not surprising ^17^. Hence, it may be challenging or impossible to develop a cord-blood specific model with acceptable accuracy. Nonetheless, Models 2 and 3 still demonstrated some clinical utility in estimating GA using cord blood samples.”*

5. Performing and reporting k-fold cross-validation for the Canadian cohort would reassure that the results are not dependent on a single randomly chosen training/testing set. The resulting models could be used for the external cohorts as well to evaluate the sensitivity of the results to the training set.

**We agree that using cross-validation/bootstrap validation approaches are often more efficient than a train/test split. And in smaller datasets it would be a concern whether the validation results were sensitive to the resultant randomly partitioned test set. However, we had the benefit of massive amounts of training data, so we opted to randomly partition the data, which simplified the validation process and reduced computational time and bandwidth required for model fitting etc. We have previously conducted a large number of sensitivity analyses including a demonstration that models and our findings have remained robust when trained and tested in different random splits of the data, and we have also trained models using a cross-validation approach, where we observed near identical model performance. Hence, we have continued to use our train/test data splitting approach with high confidence in its robustness.**

6. Please provide references and discuss the relationship between the screening analytes and the GA. For example, studies on animal models showing how each of the analytes changes as a function of GA (do they increase/decrease, are the relationships linear?)

**Although we agree with the reviewer that these details are important for understanding the underlying biochemistry of the association between analytes and GA, they are beyond the scope of this manuscript to elaborate on these in much detail. We have referred readers to our previous papers where we discussed the quantitative and qualitative nature of the associations of analytes with GA:**

*“Our research team has previously investigated the distribution of analyte levels obtained from newborn screening data in Ontario infants according to GA at birth and found that many analytes included in the newborn screening panel varied with level of prematurity, including a number of amino acids, acylcarnitines and fatty acid oxidation markers, 17-OHP, thyroid stimulating hormone and the relative proportions of fetal and adult hemoglobin subtypes ^12,13^.”*

7. Considering the small standard deviation of the GA in all of the cohorts (~1.5-2 weeks), accurate estimation within 2 weeks does not seem to be a good criterion for a successful prediction.

**We agree with the reviewer and have removed 2-week accuracy from the results.**

8. These statements in the discussion are vague. Please provide quantitative details.
- “we noted improvements in the accuracy of GA estimates overall”
- “These findings were largely consistent across the internal and external validation cohorts with some variation”

**We have revised the discussion as follows:**

*Direct comparisons of model performance across populations and between our current ML GA estimation models and previously reported conventional regression models, is challenging due to different distributions of GAs and birth weights, and other infant- and setting-specific factors. However, comparing overall internal validation results among previously published models and those presented in this manuscript, we noted improvements in the accuracy of GA estimates overall (RMSE=1.06 and 1.04 vs. 0.89 for current Model 3), and in preterm infants (RMSE=1.78 and 1.35 vs. 1.16 for current Model 3)^13,14^. These findings were largely consistent across the internal and external validation cohorts. Therefore, our interpretation is that benefits of our ML approach were clear and clinically relevant, but represented incremental improvements, suggesting that our previous models were robustly developed.*

Reviewer #2: I like this study, except for a few things. It's hard to understand how hospitals in Zambia don't have access to ultrasound, but have access to complex metabolic panels. Could the publication be framed to show how the innovation in analysis is key. You are touching at the inability to properly model GA in other countries based on Canadian data. No surprise, not an easy task. You were able to improve model prediction by including some additional variables, this is valuable.

**Our study was an external validation of a promising proof of concept non-invasive postnatal GA estimation algorithm. We agree that full implementation/scaling of such an algorithm would require the availability of national or regional reference laboratories which are often lacking in low-income settings, where there may also not be universal access to early pregnancy ultrasound/adequate prenatal care.**

In my opinion, this is a 1-5 author publication, how are you going to explain how 23 authors contributed. Bottom line, I'm not sure how valuable this publication is, other than to the 23 coauthors. No data? If there is access, it isn't clear in the publication or I missed it.

**All authors meet the requirements for authorship; however, we take the point that 23 is a lot. Although the project was led from Ottawa Ontario, it involved two international sites with research and nursing staff to coordinate the project, enroll and follow patients, as well as collect, store and ship blood samples. We feel strongly that all contributing authors should be recognized for their important contributions. We have provided a detailed contributions statement in the paper and it was also provided in the online system. We hope this is acceptable.**

While revising your submission, please upload your figure files to the Preflight Analysis and Conversion Engine (PACE) digital diagnostic tool, <https://pacev2.apexcovantage.com/>. PACE helps ensure that figures meet PLOS requirements. To use PACE, you must first register as a user. Registration is free. Then, login and navigate to the UPLOAD tab, where you will find detailed instructions on how to use the tool. If you encounter any issues or have any questions when using PACE, please email PLOS at [figures@plos.org](mailto:figures@plos.org). Please note that Supporting Information files do not need this step.
